# Conocarpan and Eupomatenoid‐6 as Natural Prototypes for Antituberculosis Compounds

**DOI:** 10.1155/ijm/9147877

**Published:** 2026-06-11

**Authors:** Lincoln Luís Silva, Anderson Valdiney Gomes Ramos, Debora Cristina Baldoqui, Nathally Claudiane de Souza Santos, Vera Lucia Dias Siqueira, Katiany Rizzieri Caleffi-Ferracioli, Rosilene Fressatti Cardoso, Rosi Zanoni da Silva, Regiane Bertin de Lima Scodro

**Affiliations:** ^1^ Department of Pharmacy, State University of Midwest Paraná, Guarapuava, Brazil; ^2^ Department of Chemistry, State University of Maringá, Maringá, Brazil, uem.br; ^3^ Department of Clinical Analysis and Biomedicine, State University of Maringá, Maringá, Brazil, uem.br; ^4^ Department of Pharmaceutical Sciences, State University of Ponta Grossa, Ponta Grossa, Brazil, uepg.br

**Keywords:** antitubercular agents, cytotoxicity, *Mycobacterium tuberculosis*, neolignans, *Piper*

## Abstract

**Background and Aim:**

Tuberculosis (TB) is caused by *Mycobacterium tuberculosis* (*Mtb*) and is the leading cause of death from a single bacterium. In addition, the increasing incidence of multidrug‐resistant tuberculosis (MDR‐TB) has led to the research of new compounds that are effective against Mtb. Therefore, in this study we evaluated the in vitro cytotoxicity and activity of two neolignans—conocarpan (1) and eupomatenoid‐6 (2)—isolated from *Piper solmsianum*, against drug‐susceptible and multidrug‐resistant (MDR‐TB) *Mtb* isolates, as well as other bacteria.

**Experimental Procedure:**

Compounds were obtained through high‐performance liquid chromatography. The in vitro activities were determined by the resazurin microtiter assay, and their combinatory effect with anti‐TB and antiretroviral were determined by the resazurin drug combination microtiter assay against *Mtb* H37Rv and 14 clinical isolates (including 10 MDR‐TB). The cytotoxicity was evaluated in J774.A1, HeLa, VERO, and MRC‐5 cells by MTT assay. The analysis of the absorption, distribution, metabolism, and excretion of neolignans was performed on Swiss ADME.

**Key Results:**

Compounds (1) and (2) showed activity against *Mtb* H37Rv, 14 Mtb clinical isolates, *Mycobacterium abscessus*, and *Staphylococcus aureus*, with the minimum inhibitory concentrations ranging from 3.90 to 125 *μ*g/mL. Only (1) exhibited synergism with rifampicin, and none of them presented an antagonistic effect. Both substances were more selective against bacteria, and (2) was the least cytotoxic. The (1) and (2) have high gastrointestinal permeability and do not violate the Lipinski rules.

**Conclusions and Implications:**

The synergistic interaction between conocarpan and rifampicin, coupled with the lack of antagonism with antiretroviral drugs, suggests that these neolignans are promising candidates for further development in the treatment of drug‐resistant TB and TB/HIV coinfection.

## 1. Introduction

Tuberculosis (TB) is a disease caused by *Mycobacterium tuberculosis* (*Mtb*), which is one of the top 10 causes of death worldwide and the leading cause of death from a single infectious agent (ranking above HIV/AIDS) [1]. Globally, an estimated number of 10.6 million cases of TB resulted in 1.3 million deaths in 2022, including 167,000 deaths among HIV‐positive people. Additionally, 410,000 cases of multidrug‐resistant tuberculosis (MDR‐TB) were reported in the same year [1]. This situation leads to the possibility of an untreatable global epidemic of TB [2]. Therefore, there is an urgent need for the discovery of highly potent and safe anti‐TB treatments [3].

Natural products have been used for the development of new drugs [4] and a lot of people depend on natural products as a primary substance for the treatment of several diseases [5]. Piperaceae family is divided into five genera, among which the genus *Piper* is the one of the greatest commercial, economic, and medicinal importance [6]. *Piper* plants are widely distributed in pantropical regions where many of them have been used to treat urological, skin, liver, and stomach disorders [7]. Metabolites isolated from *Piper* species show a wide range of activity such as anticancer, antiviral, antibacterial, anti‐TB, antifungal, leishmanicidal, and larvicidal [8, 9].

Previous phytochemical studies using *Piper solmsianum* indicated the presence of dihydrobenzofuran and tetrahydrofuran neolignans [10, 11]. While the antitubercular activity of some neolignans, such as eupomatenoid‐5 and conocarpan, was reported [9], the specific activity of eupomatenoid‐6 (2) against *M. tuberculosis* remained undetermined. Additionally, important questions regarding performance against multidrug‐resistant (MDR) clinical isolates, their potential synergistic effects with first‐line drugs like rifampicin (RIF), and their safety profile when coadministered with highly active antiretroviral therapy (HAART) have not yet been addressed. Taking into account the urgent need in the search for new drugs against TB, this study provides a comprehensive pharmacological evaluation of conocarpan (1) and eupomatenoid‐6 (2), isolated from *P. solmsianum*, positioning these neolignans not merely as isolated active metabolites, but as viable scaffolds for TB/HIV coinfection therapy.

## 2. Methods

### 2.1. Plant Material

Aerial parts of *P. solmsianum* were collected in Ponta Grossa, Paraná, Brazil, S25°05 ^′^38 ^″^, W50°09 ^′^30 ^″^, in May 2013. Dr. Elsie Franklin Guimarães executed the botanical identification, and a voucher was registered as RB 368597 in the Herbarium at the Botanical Garden of Rio de Janeiro, Rio de Janeiro, Brazil. The crude extract was kindly provided by Prof. Ph.D. Rosi Zanoni da Silva, from State University of Ponta Grossa, Paraná, Brazil. The extraction was carried out as described by Campos et al. [12]. This research is registered in the National System for the Management of Genetic Heritage and Associated Traditional Knowledge (SisGen) Number AB486FE.

### 2.2. Isolation, Purification, and Identification of Compounds

The crude extract (1 g) was dissolved in methanol/water (1:1 50 mL, v/v), and partitioned with n‐hexane and dichloromethane. An aliquot of dichloromethane fraction (250 mg) was submitted to silica gel 60 column chromatography (0.063–0.200 mm) 70–230 mesh (Merck Millipore) eluted with hexane and ethyl acetate in concentrations from 2% up to 10% of ethyl acetate, giving Subfractions F‐1–F‐60. Later, thin layer chromatography was performed on silica gel 60GF_254_ plates (Merck, Darmstadt, Germany), and the compounds were visualized under the UV light (250–360 nm) and after spraying a solution of vanillin‐sulfuric acid followed by heating the plates to 100°C for 5 min. Fraction 48 was identified as eupomatenoid‐6 (**2**) and fraction 54 as conocarpan (**1**) by analyses of spectral data of ^1^H nuclear magnetic resonance (NMR). NMR spectra were recorded on a Bruker spectrometer (Bruker BioSpin, Billerica, Massachusetts, United States), operating at 300 MHz for ^1^H, using deuterated chloroform (CDCl_3_) and tetramethylsilane (TMS) (Merck, Germany) as the internal standard. The data were visualized on MestReNova (Version 12.0.1‐20560) and compared with the literature [9, 13, 14]. The ^1^H NMR spectra for Neolignans (1) and (2) are provided in File S1.

**Figure 1:**
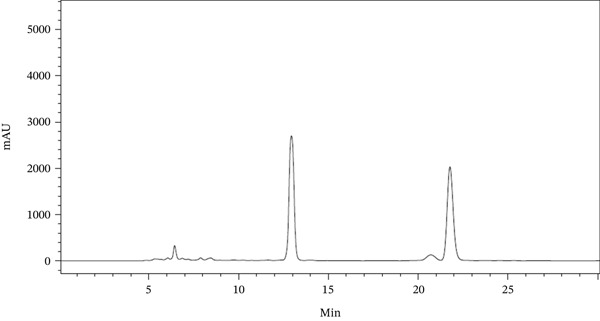
Chromatogram obtained after injection of subfractions of *Piper solmsianum*. Analysis conditions: 85% methanol, flow rate 12 mL/min; at *λ* = 280 nm. Conocarpan (1) with the retention time at 12.5 min and eupomatenoid‐6 (2) at 21.3 min.

Fractions 41–56 were filtered and submitted to semipreparative HPLC purification (Shimadzu, Prominence, LC‐20AR) equipped with a column Shim‐pack PREP‐ODS C18 (250 × 20 mm; 15 *μ*m) and a diode array detector (SPD‐M20A). The separation was carried out in an isocratic system using as mobile phase a mixture of methanol (Merck, Millipore) and water (Milli‐Q system, Millipore, Burlington, Massachusetts, United States) (85:15, v/v), with a flow rate of 12.0 mL/min. The detection was carried out at 280 nm, and the running time was 30 min, affording 23 mg of Compound (**1**) and 16 mg of (**2**). The HPLC results were compared with the literature (Figure 1) [15]. The purity of the isolated neolignans was determined to be > 95% for both conocarpan (1) and eupomatenoid‐6 (2), as calculated by the normalization of the peak areas in the HPLC chromatograms (*λ* = 280 nm).

Compound (**1**): Conocarpan.

4‐[(2R,3R)‐3‐methyl‐5‐[(E)‐prop‐1‐enyl]‐2,3‐dihydro‐1‐benzofuran‐2‐yl]phenol, white solid, ^1^H NMR (300 MHz, CDCl_3_): *δ* 7.26 (2H, dd, J = 8.4, 2.0 Hz, H‐2 ^′^and H‐6 ^′^), 7.12 (1H, d, J = 8.1 Hz, H‐6), 7.11 (1H, sl, H‐4), 6.84 (2H, dd, J = 8.6, 2.0 Hz, H‐3 ^′^ and H‐5 ^′^), 6.76 (1H, d, J = 8.1 Hz, H‐7), 6.37 (1H, dd, J = 15.7, 1.5 Hz, H‐8); 6.09 (1H, dq, J = 15.6 and 6.5 Hz, H‐9); 5.08 (1H, d, J = 8.8 Hz, H‐2); 4.89 (s, OH‐4 ^′^); 3.40 (1H, m, H‐3); 1.86 (3H, dd, J = 6.6 e 1.6 Hz, CH_3_‐10); 1.39 (3H, d, J = 6.8 Hz, CH_3_‐3). HPLC retention time at 12.5 min.

Compound (**2**): Eupomatenoid‐6.

4‐[3‐methyl‐5‐[(E)‐prop‐1‐enyl]‐1‐benzofuran‐2‐yl]phenol, white solid, ^1^H NMR (300 MHz, CDCl_3_): *δ* 7.69 (2H, dd, J = 8.8, 2.0 Hz, H‐2 ^′^ and H‐6 ^′^), 7.44 (1H, d, J = 1.6 Hz, H‐4), 7.37 (1H, d, J = 8.4 Hz, H‐7), 7.28 (1H, dd, J = 1.6, 8.4 Hz, H‐6), 6.94 (2H, dd, J = 8.8, 2.1 Hz, H‐3 ^′^ and H‐5 ^′^), 6.52 (1H, dd, J = 15.7 e 1.5 Hz, H‐8); 6.23 (1H, dq, J = 15.6 e 6.6 Hz, H‐9); 5.03 (1H, s, OH‐4 ^′^); 2.43 (3H, s, CH_3_‐3); 1.91 (3H, dd, J = 6.6 e 1.6 Hz, CH_3_‐10). HPLC retention time at 21.3 min.

### 2.3. Bacteria and Growth Conditions

*Mtb* H37Rv (ATCC 27294) and 14 *Mtb* clinical isolates (2 susceptible, 1 isoniazid (INH)‐resistant, 1 streptomycin‐resistant, and 10 MDR) were cultured in Middlebrook 7H9 broth medium (Difco Laboratories, Detroit, United States), supplemented with 10% oleic acid, bovine albumin, dextrose and catalase (OADC) enrichment (BBL/Becton‐Dickinson, Sparks, Maryland, United States), and 0.02% glycerol (Sigma‐Aldrich, St. Louis, Missouri, United States) (7H9‐OADC), for 15–21 days at 35°C.

Nontuberculous mycobacteria (NTM) of clinical interest were grown in Müeller–Hinton Broth (Difco Laboratories, Detroit, United States) cation‐adjusted (CAMHB) with calcium (Ca^+2^) and magnesium (Mg^+2^), according to the Clinical and Laboratory Standards Institute [16]. Culture for slowly growing NTM, such as *Mycobacterium avium*, was supplemented with 10% of OADC for 7 days at 35°C, and rapidly growing NTM like *Mycobacterium abscessus* (ATCC 19977) was cultured in CAMHB for 5 days at 30°C [17].

*Staphylococcus aureus* (ATCC 29213), *Enterococcus faecalis* (ATCC 29212), and *Escherichia coli* (ATCC 25922) were grown in Müeller–Hinton agar (Difco Laboratories, Detroit, United States) for 24 h at 35°C.

### 2.4. Minimum Inhibitory Concentration (MIC)

The activities of (**1**) and (**2**) against *Mtb* were evaluated in triplicate on independent days by resazurin microtiter assay (REMA) plate [9, 18, 19]. Stock solutions of (**1**) and (**2**) at 10,000 *μ*g/mL were prepared in dimethyl sulfoxide (DMSO, Sigma‐Aldrich, St. Louis, Missouri, United States) and twofold serial dilutions in 7H9‐OADC, directly in 96‐well microplate were carried out to obtain final concentrations ranging from 0.98 to 250 *μ*g/mL. The mycobacterial growth was adjusted to OD_625_ (0.16–0.20), equivalent to McFarland Scale 1 (3 × 10^8^ CFU/mL), and then diluted 1:20 in 7H9‐OADC. In the sequence, 100 *μ*L of standardized inoculum was added to each well of the microplate containing 100 *μ*L of compounds in their respective concentrations. The microplates were covered and incubated at 35°C, in a normal atmosphere, for 7 days. After this period, 30 *μ*L of freshly prepared 0.02% resazurin solution (Sigma‐Aldrich St Louis Missouri, United States) was added to each well and the microplates were reincubated at 35°C for an additional 24–48 h. A color change from blue to pink indicated mycobacterial growth, and the MIC was interpreted as the lowest compound concentration that prevented the color change. Medium, compound sterility, bacterial growth with and without 2.5% (v/v) DMSO controls were included in all assays. INH (Sigma‐Aldrich, St. Louis, Missouri, United States) was used as the reference drug at concentrations from 0.007 to 1.0 *μ*g/mL.

The spectrum of activity against gram‐positive and gram‐negative bacteria was performed following the CLSI M07‐A10 protocol, and M24‐A2 for NTM, in triplicate on independent days. The previously prepared stock solution of the Compounds (**1**) and (**2**) was serially diluted, ranging from 250 to 0.98 *μ*g/mL in 96‐well microplate containing CAMHB. Bacterial growth was standardized according to McFarland 0.5 scale (OD_625_ = 0.08–0.12) and diluted in CAMHB 1:20 for gram‐positive and gram‐negative, and 1:300 for NTM [16, 17]. Next, 20 *μ*L of each standardized bacterial inoculum was added to each well of the microplate containing compounds dilutions. Gram‐positive and gram‐negative bacteria were incubated for 16–20 h at 35°C, *M. abscessus* for 3 days at 30°C, and *M. avium* for 5 days at 35°C.

After the incubation period, a solution of 1% of 2,3,5‐triphenyltetrazolium chloride (TTC, Inlab, São Paulo, Brazil) was prepared, and 10 *μ*L were added to each well of gram‐positive and gram‐negative bacteria, and the plates were reincubated for an additional 30 min at 35°C. The MIC was interpreted as the lowest compound concentration that prevented color change from colorless to red. Regarding the NTMs MIC reading, 30 *μ*L of freshly prepared 0.02% resazurin solution was added to the plates and reincubated for an additional 24 h. The MIC was interpreted in the same way as for *Mtb*. Bacterial growth, medium, and drug sterility controls were included in all assays, and ciprofloxacin was used as drug control.

### 2.5. Combined Treatment With Drugs

The activity of Compounds (**1**) and (**2**) in combination with anti‐TB and antiretroviral drugs against *Mtb* H37Rv was performed following the resazurin drugs combination microtiter assay (REDCA) [20]. Compounds (**1**) and (**2**) were tested in combination with anti‐TB drugs as INH, RIF, ethambutol (EMB), pyrazinamide (PZA), and antiretroviral drugs such as nevirapine (NVP, Fundação Oswaldo Cruz/Farmanguinhos, Brazil), atazanavir (AZV, Bristol‐Myers Squibb, New York, United States), and tenofovir disoproxil (TNV, Blenver Farmoquímica e Farmacêutica S.A, Brazil), kindly provided by the Paraná Medication Center (CEMEPAR), Paraná State Public Health Secretariat, Brazil. The assessment of fractional inhibitory concentration index (FICI) was calculated using the formula FICI = (MIC A + neolignan/MIC A) + (MIC neolignan + A/MIC neolignan), in which MIC A + neolignan represents the MIC of Drug A associated with the neolignan. MIC neolignan + A represents the MIC of neolignan associated with Drug A. MIC A and MIC neolignan represent the MIC of Drugs A and neolignan tested separately. FICI was considered synergism (FICI ≤ 0.5), indifferent (0.5 > FICI ≤ 4.0) or antagonism (FICI > 4.0).

### 2.6. Cytotoxicity Assay

The evaluation of cytotoxicity effect caused by the Compounds (**1**) and (**2**) was performed using the MTT [3‐(4,5‐dimethylthiazol‐2‐yl)‐2,5‐diphenyltetrazolium bromide] assay on the cells lineage: VERO (ATCC CCL81), HeLa (ATCC CL‐2), macrophages (J774.A1 ATCC TIB‐67), and MRC‐5 (ATCC CL‐171) [21]. VERO, HeLa, and MRC‐5 cells were cultured in Dulbecco′s Modified Eagle′s Medium (DMEM, Sigma‐Aldrich, St. Louis, Missouri, United States), and macrophages in RPMI‐1640 medium with l‐glutamine (Sigma‐Aldrich, St. Louis, Missouri, United States), both supplemented with 10% fetal bovine serum (FBS, Gibco, Life Technologies, Paisley, United Kingdom), penicillin (100 U/mL), and streptomycin (100 *μ*g/mL) (Gibco, Life Technologies, Paisley, United Kingdom). Cells were plated at a density of 5 × 10^4^ cells/well into 96‐well plate and incubated at 37°C in a humidified CO_2_ incubator (5% CO_2_) for 24 h. After that, the old medium was replaced by a new one with compounds diluted starting from 250 to 3.12 *μ*g/mL, and then the plates were incubated for 24 h. Concomitantly, another assay following the same conditions was performed with 72 h of incubation to verify potential cytotoxicity effect based on the United States National Cancer Institute plant screening program criteria [22, 23]. After this period, the wells were washed with phosphate‐buffered saline (PBS), and 50 *μ*L of MTT dissolved in PBS (2 *μ*g/mL) was added to the wells. The plates were incubated under the same conditions for 3 h, protected from the light. Later, the supernatant was discarded and the precipitate was resuspended in 150 *μ*L of DMSO to dissolve the formazan crystal. For the quantification of living cells, absorbances were read at 550 nm on a microplate reader (Spectramax Plus 384 Microplate Reader, Molecular Devices, Sunnyvale, California, United States). The half‐maximum inhibitory concentration (IC_50_) was defined as the concentration capable of reducing cell viability to 50% compared with untreated cells, and the selectivity index (SI) was determined by the (IC_50_)/(MIC) ratio [21].

Red cell toxicity was determined by the hemolysis assay, as described by [24]. A 6% red cell suspension was prepared using defibrinated sheep′s blood with 5% sterile glycosylated saline. The 100 *μ*L microliters of cell suspension were added into the wells containing 100 *μ*L of compounds previously diluted from 250 to 0.24 *μ*g/mL. A total of 100 mL of Triton X‐100 (Merck, Darmstadt, Germany) 4% were used as the positive control, whereas 100 *μ*L of untreated cells were used as a negative control. The plates were incubated at 37°C for 2 h in a humidified CO_2_ incubator (5% CO_2_). Next, the plates were centrifuged at 1000 g for 10 min, and the supernatant was transferred to a new plate, whose absorbance was read at 550 nm. The evaluation of the cytotoxicity in erythrocytes was calculated using the equation to obtain the percentage of hemolysis, in which Ap stands for positive control absorbance, As: compounds absorbance, and Ac: controls absorbance: Hemolysis (*%*) = 100 − [(Ap − As)/(Ap − Ac)] × 100. The results are reported as the concentration of compounds that caused hemolysis in 50% of erythrocytes (HC_50_).

### 2.7. Swiss ADME

Drug development involves assessment of absorption, distribution, metabolism, and excretion (ADME). In order to predict pharmacokinetics based on computer models, this study accessed the Swiss ADME, a web tool presented as freely accessible that provides important parameters used for drug development such as lipophilicity, pharmacokinetics, and drug‐likeness [25].

## 3. Results

**Figure 2:**
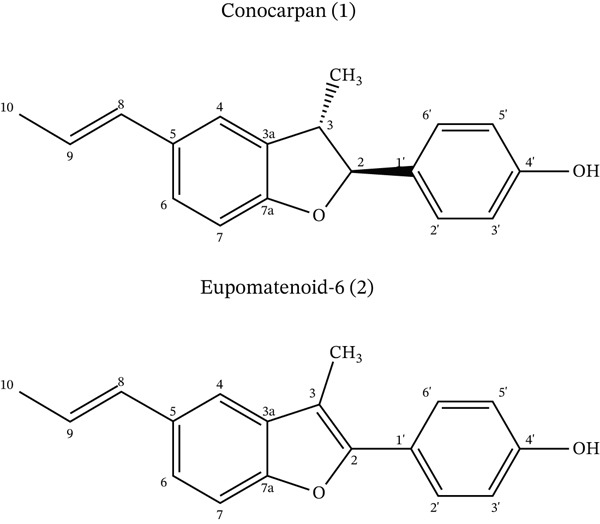
Chemical structures of the bioactive neolignans isolated from the leaves of *Piper solmsianum*: conocarpan (1) and eupomatenoid‐6 (2). These compounds were identified as the major constituents responsible for the antituberculosis activity evaluated in this study.

The purification of *P. solmsianum* extract resulted in the isolation of conocarpan (**1**) and eupomatenoid‐6 (**2**) (Figure 2).

The anti‐*Mtb* activity of (**1**) and (**2**) was assayed, and the results of MIC against *Mtb* H37Rv are shown in Table 1. The Compounds (**1**) and (**2**) showed the same activity against the *Mtb* H37Rv (15.62 *μ*g/mL). The neolignans were also tested against 14 *Mtb* clinical isolates with different drug resistance profiles (Table 1). The MICs for the *Mtb* clinical isolates ranged from 7.81 to 62.5 *μ*g/mL for Compound (**1**) and from 7.81 to 125 *μ*g/mL for Compound (**2**).

**Table 1:** Conocarpan (1) and eupomatenoid‐6 (2) minimum inhibitory concentration (MIC) against *Mycobacterium tuberculosis.*

*Mtb* strain/isolate	Drug susceptibility	MIC *μ*g/mL/*μ*M		
		Isoniazid	(1)	(2)
H37Rv	Susceptible	0.06/0.44	15.62/58.66	15.62/59.1
36	Susceptible	0.06/0.44	15.62/58.66	15.62/59.1
49	Susceptible	0.03/0.22	15.62/58.66	15.62/59.1
BRF4	H^R^	4/29.17	15.62/58.66	125/472.94
4250	H^R^	2/14.58	15.62/58.66	7.81/29.55
BRF7	S^R^	0.03/0.22	15.62/58.66	15.62/59.1
109	H^R^, R^R^	1/7.29	15.62/58.66	7.81/29.55
309	H^R^, R^R^	1/7.29	15.62/58.66	7.81/29.55
18	H^R^, R^R^, E^R^	2/14.58	31.25/117.35	7.81/29.55
19RP	H^R^, R^R^, E^R^	**4/29.17**	**7.81/29.33**	**7.81/29.55**
64A	H^R^, R^R^, Z^R^	**8/58.33**	31.25/117.35	**15.62/59.1**
71A	H^R^, R^R^, E^R^, Z^R^	4/29.17	62.5/234.70	31.25/118.23
73A	H^R^, R^R^, Z^R^	4/29.17	31.25/117.35	31.25/118.23
1193	H^R^, R^R^, S^R^, E^R^	**4/29.17**	**7.81/29.33**	**7.81/29.55**
3614	H^R^, R^R^, S^R^, E^R^, Z^R^, Et^R^	4/29.17	31.25/117.35	15.62/59.1

*Note:* The best results compared with isoniazid are shown in bold.

Abbreviations: E, ethambutol; Et, ethionamide; H, isoniazid; R, rifampicin; ^R^, resistant; S, streptomycin; Z, pyrazinamide.

In order to evaluate the activity of these compounds in other bacteria, the MIC assay was performed in NTM and gram‐positive and gram‐negative bacteria. Compound (**1**) showed to have activity against *M. abscessus* (MIC 7.81 *μ*g/mL), but the same did not occur with Compound (**2**) (MIC 125 *μ*g/mL). For slowly growing NTM (*M. avium*), Compound (**1**) showed better activity (31.25 *μ*g/mL) when compared with eupomatenoid‐6 (> 250 *μ*g/mL) (Table 2). The assay to determine the MIC of (**1**) and (**2**) against gram‐positive (*S. aureus* and *E. faecalis*) and gram‐negative (*E. coli*) bacteria showed a value > 250 *μ*g/mL in *E. coli* and *E. faecalis*. On the other hand, Compounds (**1**) and (**2**) were active against *S. aureus* with MIC 7.81 and 3.9 *μ*g/mL, respectively.

**Table 2:** Conocarpan (**1**) and eupomatenoid‐6 (**2**) minimum inhibitory concentration (MIC) against nontuberculous mycobacteria, gram‐positive, and gram‐negative bacteria.

Bacteria	MIC *μ*g/mL/*μ*M	
	(1)	(2)
*Mycobacterium abscessus*	7.81/29.32	125/472.95
*Mycobacterium avium*	31.25/117.35	> 250/> 945.9
*Staphylococcus aureus*	7.81/29.32	3.90/14.75
*Enterococcus faecalis*	> 250/> 938.8	> 250/> 945.9
*Escherichia coli*	> 250/> 938.8	> 250/> 945.9

The combination of Compounds (**1**) or (**2**) with drugs used in the treatment of TB and HIV were evaluated and are shown in Table 3. It is noteworthy that (**1**) showed synergism with RIF against *Mtb* H37Rv (FICI = 0.37). Interestingly, no antagonism was observed with Compounds (**1**) or (**2**) when combined with RIF, INH, PZA, EMB, NVP, AZV, and TNV.

**Table 3:** Conocarpan (**1**) and eupomatenoid‐6 (**2**) minimum inhibitory concentration (MIC) and combinatory activity with antituberculosis and antiretroviral drugs against *Mycobacterium tuberculosis* H37Rv.

Compound	MIC *μ*g/mL	FICI						
		INH	EMB	RIF	PZA	NVP	AZV	TNV
(1)	15.62	1.00	0.62	**0.37**	1.00	1.00	0.75	2.00
(2)	15.62	2.00	0.75	0.75	0.56	0.75	2.00	2.00

*Note:* Bold value denotes synergism.

Abbreviations: AZV, atazanavir; EMB, ethambutol; FICI, fractional inhibitory concentration index; INH, isoniazid; NVP, nevirapine; PZA, pyrazinamide; RIF, rifampicin; TNV, tenofovir disoproxil.

Computational analysis via Swiss ADME was used to determine the drug‐likeness and pharmacokinetic profile of Neolignans (**1**) and (**2**) (Table 4). Both compounds fulfilled all Lipinski Rule of Five criteria, indicating a high probability of oral bioavailability. A key finding was the low topological polar surface area (TPSA = 29.46 Å^2^) and the calculated lipophilicity (log Po/w 3.91–4.29), parameters that are associated with high passive membrane permeability and gastrointestinal absorption. Regarding drug–drug interaction potential, neither compound was predicted as an inhibitor of the CYP3A4 isoenzyme, which is responsible for the metabolism of most antiretroviral drugs used in HAART. However, Compound (1) showed a broader inhibition profile for other isoforms (CYP2C9 and 2D6), whereas Compound (2) did not inhibit these enzymes, suggesting a more selective pharmacokinetic safety profile.

**Table 4:** Computational analysis of absorption, distribution, metabolism, and excretion of conocarpan (**1**) and eupomatenoid‐6 (**2**) by Swiss ADME.

Compounds	(1)	(2)
HBA	2	2
HBD	1	1
TPSA (Å^2^)	29.46	29.46
Lipophilicity log Po/w	3.91	4.29
Water solubility class	Moderately soluble	Poorly soluble
GI absorption	High	High
BBB permeant	Yes	Yes
CYP1A2 and CYP2C19 inhibitors	Yes	Yes
CYP2C9 and CYP2D6 inhibitors	Yes	No
CYP3A4 inhibitor	No	No
Lipinski violations	0	0

Abbreviations: ADME, absorption, distribution, metabolism, and excretion; BBB, blood–brain barrier; GI, gastrointestinal absorption; HBA, hydrogen bond acceptors; HBD, hydrogen bond donors; TPSA: topological polar surface area.

The SI was calculated for all cell lineages tested and it is shown in File S2. Overall, the SI for the Compound (**1**) ranged from 0.03 to 5.30, whereas the Compound (**2**) ranged from 0.07 to 17.51, considering all bacteria tested. In addition, both compounds maintained their IC_50_ above 4 *μ*g/mL after exposing the cells to the neolignans for 72 h. Besides that, (**1**) and (**2**) presented low hemolytic activity up to 250 *μ*g/mL.

## 4. Discussion

Considering the alarming progress of TB in the world, there is an urgent need for the discovery of novel, potent, and safe TB treatments [3]. Therefore, natural products might play a significant role in the discovery and development process of new drugs [26]. As seen in the previous study conducted by our research group, which introduced neolignans having notorious activity against *Mtb* [9], this study corroborates, complements, and reinforces the potential of these molecules against the *Mtb*. Our results showed that the Compounds (**1**) and (**2**) were in the leaves of *P. solmsianum*, and the chromatogram is in accordance with previous studies [15]. Moreover, these compounds showed auspicious activities as anti‐*Mtb* agents, inhibiting bacillary growth at low concentrations.

Compounds (**1**) and (**2**) are structurally similar and showed MIC of 15.62 *μ*g/mL against *Mtb* H37Rv, and also presented a similar MIC of 86% *Mtb* clinical isolates. Interestingly, the Compounds (**1**) and (**2**) showed lower MIC against *Mtb* H37Rv compared with PZA (100 *μ*g/mL or 812.15 *μ*M), the drug that is already used for the treatment of TB. Besides, (**1**) and (**2**) exhibited similar activities as terizidone (MIC 8–32 *μ*g/mL or 26.46–105.86 *μ*M), a drug usually used in the treatment of MDR‐TB [27].

Scodro et al. [9] verified that eupomatenoid‐5, a neolignan with hydroxyl and methoxy groups in positions 4 ^′^ and 5 ^′^, respectively, obtained a MIC of 1.9 *μ*g/mL (or 6.45 *μ*M) also against *Mtb.* Moreover, Campos et al. [12] suggested that the presence of a hydroxyl group at the 4 ^′^ position has a more important role in the antibacterial activity. The structure of benzofurans has also been studied by [28] in the antifungal activity and suggested that the absence of a methoxy group at the 3 ^′^ position of the phenyl‐propenyl‐benzofuran plays a critical role. For all that, this important hydroxyl group at the 4 ^′^ position was also observed in the Compounds (**1**) and (**2**). Additionally, (**1**) exhibited synergism with RIF, lowering the MIC of (**1**) from 15.62 to 1.95 *μ*g/mL (7.13 *μ*M). This synergistic effect resulted in an inhibitory activity comparable to the MIC previously reported for eupomatenoid‐5 (1.9 *μ*g/mL) [9]. Therefore, we can speculate that the bond at the position 2,3 of phenyl‐propenyl‐benzofuran could have contributed to the synergism. Thus, (**1**) may be an alternative against TB and further studies should clarify this applicability in the TB treatment.

Effective treatment of TB relies on drugs administered in combination to guarantee antimicrobial efficacy while preventing the selection of drug‐resistant mutants to a single drug and achieve cure [29]. In this sense, anti‐*Mtb* new medicines should be safe, show no antagonism with other antimicrobials, decrease the required treatment time and improve the outcomes [30]. Moreover, new compounds should not be antagonistic with antiretroviral therapy because there is a high potential for drug interactions in anti‐HIV and anti‐TB therapy [30, 31]. The antiretroviral NVP, AZV, and TNV did not show antagonism with (**1**) nor (**2**) against *Mtb* H37Rv. This finding is particularly relevant because it was not explored in previous studies of these neolignans [9], addressing one of the main obstacles for the clinical use of new anti‐TB candidates in coinfected patients under HAART. Thus, thinking of the treatment for TB/HIV coinfection, the Compound (**2**) should be considered for further studies, because Swiss ADME demonstrated that (**2**) does not inhibit CYP2C9, CYP2D6, and CYP3A4, important enzymes of CYP450 complex that are of great concern in antiretroviral therapy [31].

Regarding the spectrum of activity, it is preferable that an anti‐TB drug candidate has a narrow spectrum of activity to *Mtb*, without interfering in the human microbiota [26, 32]. Here, neither (**1**) nor (**2**) were active against *E. faecalis* and *E. coli*, common species found in the guts. On the other hand, the two neolignans were active against *S. aureus* and *M. abscessus*. In other studies, (**2**) was also highly effective against *Candida glabrata* [33], and (**1**) turned out to be active against *Trypanosoma cruzi* [34]. Although (**1**) and (**2**) were active against other pathogens than *Mtb*, this should not be an obstacle once RIF also has broad‐spectrum activity and is a first‐line anti‐TB drug [35].

The development of new drugs requires detailed information on their safety and toxicity [29]. One way to evaluate these variables is to study the effects on cells using in vitro cytotoxicity assay. In this study, different cell lines were used, and the SI was determined for each one. The SI can be considered as a preliminary estimation of the therapeutic window [30]. The SI values for (**1**) in J774.A1 cells ranged from 0.61 to 4.88, similar to what was found by Scodro et al. [9], who evaluated (**1**) on macrophages (J774.G8) and obtained a SI of 6.5. Overall, the Compound (**2**) showed the lowest cytotoxic effect in all cell lineages assayed.

It is noteworthy that, according to the United States National Cancer Institute plant screening program, a pure compound with IC_50_ value ≤ 4 *μ*g/mL is generally considered cytotoxic [22, 36]. Therefore, these neolignans are considered noncytotoxic compounds since the IC_50_ values remained above 4 *μ*g/mL after exposing the cells to the compounds for 72 h.

Cytotoxicity assays should be followed by the red blood cell hemolysis assay, which provides a simple and effective means of eliminating molecules that cause damage in mammalian cell membranes [37]. Studies on the hemolytic activity of new compounds are essential to guarantee their use since some drugs may cause adverse effects such as hemolytic anemia induced by antimicrobials, especially [38]. Notably, (**1**) and (**2**) showed very low hemolytic activity (< 1% hemolysis) in a concentration lower than 250 *μ*g/mL, or a concentration almost 16‐folds higher than the MIC value.

The Swiss ADME analysis has given important information about the two assayed molecules. One of the most important factors to be taken into account is Lipinski′s five rules, which evaluate if a compound is more likely to be active in humans. The five rules state that poor absorption is more likely when: there are more than five H‐bond donors, the molecular weight is over 500 g/mol, the partition coefficient *log P* is over 5, and there are more than 10 H‐bond acceptors [39]. Both compounds do not violate those rules, and therefore, they are promising compounds for new drugs against TB.

The interest in herbal medicine remains a substantial element of healthcare systems [40]. Here, it was shown the study of two neolignans against *Mtb*, one of the deadliest microorganisms in the world. The Compounds (**1**) and (**2**) showed to be active against *Mtb*, including multiresistant clinical isolates showing good selectivity in the tested cells. The better spectrum of activity covered *Mtb*, *M. abscessus*, and *S. aureus*. The synergism between conocarpan (**1**) and RIF draws attention to an improvement in the treatment of TB, especially in cases of TB/HIV coinfection in which their use in combination might lead to increased treatment efficacy, shortening treatment time, and improving safety. In this sense, the neolignans may be considered promising prototypes as anti‐TB drug candidates.

## Author Contributions

L.L.S.: conceptualization, data curation, formal analysis, visualization, investigation, writing—original draft. A.V.G.R.: methodology, validation, investigation. D.C.B.: methodology, validation, investigation. N.C.S.S.: methodology, validation, investigation. V.L.D.S.: writing—review & editing. K.R.C‐F.: writing—review & editing. R.F.C.: writing—review & editing. R.Z.S.: writing—review & editing. R.B.L.S.: resources, supervision, project administration, writing—review & editing.

## Funding

No funding was received for this manuscript.

## Conflicts of Interest

The authors declare no conflicts of interest.

## Supporting information


**Supporting Information** Additional supporting information can be found online in the Supporting Information section. File S1: High‐resolution ^1^H NMR spectra (300 MHz) of neolignans conocarpan (1) and eupomatenoid‐6 (2) isolated from *Piper solmsianum.* File S2: Table containing the complete cytotoxicity data (IC50) and selectivity index (SI) for Neolignans (1) and (2) across all tested cell lineages (J774.A1, VERO, HeLa, MRC‐5, and SBC) against *M. tuberculosis* H37Rv, clinical isolates, nontuberculous mycobacteria (NTM), and gram‐positive/negative bacteria.

## Data Availability

The datasets generated/analyzed for this study can be found in the Figshare repository (10.6084/m9.figshare.26384404).
