# Supplementary material for: Conocarpan and Eupomatenoid‐6 as Natural Prototypes for Antituberculosis Compounds
Source: Int J Microbiol. 2026 Jun 11;2026:9147877. doi: 10.1155/ijm/9147877 (PMC13254484; doi:10.1155/ijm/9147877)
Supplement: Supplementary file 1 — Supporting Information Additional supporting information can be found online in the Supporting Information section. File S1: High‐resolution 1H NMR spectra (300 MHz) of neolignans conocarpan (1) and eupomatenoid‐6 (2) isolated from Piper solmsianum. File S2: Table containing the complete cytotoxicity data (IC50) and selectivity index (SI) for Neolignans (1) and (2) across all tested cell lineages (J774.A1, VERO, HeLa, MRC‐5, and SBC) against M. tuberculosis H37Rv, clinical isolates, nontuberculous mycobacteria (NTM), and gram‐positive/negative bacteria. [file IJM-2026-9147877-s001.docx]

**SUPPLEMENTARY MATERIAL**

Conocarpan and eupomatenoid-6 as natural prototypes for antituberculosis compounds

**Short Title:** Piper neolignans as antituberculosis prototypes

**Authors:**

Lincoln Luís Silva^1^, Anderson Valdiney Gomes Ramos^2^, Debora Cristina Baldoqui^2^, Nathally Claudiane de Souza Santos^3^, Vera Lucia Dias Siqueira^3^, Katiany Rizzieri Caleffi-Ferracioli^3^, Rosilene Fressatti Cardoso^3^, Rosi Zanoni da Silva^4^, Regiane Bertin de Lima Scodro^5^

**Affiliation:**

^1^Department of Pharmacy, State University of the Centre-West, Guarapuava, Paraná, Brazil;

^2^Department of Chemistry, State University of Maringá, Maringá, Paraná, Brazil;

^3^Graduate Program in Biosciences and Physiopathology, State University of Maringá, Paraná, Brazil;

^4^Department of Pharmaceutical Sciences, State University of Ponta Grossa, Paraná, Brazil;

^5^Graduate Program in Health Sciences, State University of Maringá, Maringá, Paraná, Brazil;

Contents

[Supplementary File S1: 3](#_Toc229516509)

[Supplementary File S2: 4](#_Toc229516510)

Conocarpan (**1**)

Eupomatenoid-6 (**2**)

Supplementary File S1: ^1^H NMR (300 MHz) spectra of conocarpan (**1**) and eupomatenoid-6 (**2**) purified from the leaves of *Piper solmsianum.*

| Supplementary File S2: Table containing the complete cytotoxicity data (IC50) and selectivity index (SI) for neolignans (1) and (2) across all tested cell lineages (J774.A1, VERO, HeLa, MRC-5, and SBC) against *M. tuberculosis* H37Rv, clinical isolates, Nontuberculous Mycobacteria (NTM), and gram-positive/negative bacteria. | | | | | | | | | | | | | | | | | | | | | | |
| --- | --- | --- | --- | --- | --- | --- | --- | --- | --- | --- | --- | --- | --- | --- | --- | --- | --- | --- | --- | --- | --- | --- |
|  |  | | **J774.A1** | | | | **VERO** | | | | **HeLa** | | | | **MRC-5** | | | | **SBC** | | | |
| **Microorganism** | **MIC**  **(µg/mL)** | | **IC_50_**  **(µg/mL)** | | **SI** | | **IC_50_**  **(µg/mL)** | | **SI** | | **IC_50_**  **(µg/mL)** | | **SI** | | **IC_50_**  **(µg/mL)** | | **SI** | | **HC_50_**  **(µg/mL)** | | **SI** | |
| ***Mtb*** | (**1**) | (**2**) | (**1**) | (**2**) | (**1**) | (**2**) | (**1**) | (**2**) | (**1**) | (**2**) | (**1**) | (**2**) | (**1**) | (**2**) | (**1**) | (**2**) | (**1**) | (**2**) | (**1**) | (**2**) | (**1**) | (**2**) |
| H_37_R_V_ | 15.62 | 15.62 | 38.13 | 45.17 | 2.44 | 2.89 | 41.39 | 68.29 | 2.65 | 4.37 | 6.84 | 17.31 | 0.44 | 1.11 | 22.09 | 41.27 | 1.41 | 2.64 | >250 | >250 | >16.04 | >16.04 |
| 36 | 15.62 | 15.62 | 38.13 | 45.17 | 2.44 | 2.89 | 41.39 | 68.29 | 2.65 | 4.37 | 6.84 | 17.31 | 0.44 | 1.11 | 22.09 | 41.27 | 1.41 | 2.64 | >250 | >250 | >16.04 | >16.04 |
| 49 | 15.62 | 15.62 | 38.13 | 45.17 | 2.44 | 2.89 | 41.39 | 68.29 | 2.65 | 4.37 | 6.84 | 17.31 | 0.44 | 1.11 | 22.09 | 41.27 | 1.41 | 2.64 | >250 | >250 | >16.04 | >16.04 |
| BRF4 | 15.62 | 125 | 38.13 | 45.17 | 2.44 | 0.36 | 41.39 | 68.29 | 2.65 | 0.55 | 6.84 | 17.31 | 0.44 | 0.14 | 22.09 | 41.27 | 1.41 | 0.33 | >250 | >250 | >16.04 | >2.00 |
| 4250 | 15.62 | 7.81 | 38.13 | 45.17 | 2.44 | **5.78** | 41.39 | 68.29 | 2.65 | **8.74** | 6.84 | 17.31 | 0.44 | **2.22** | 22.09 | 41.27 | 1.41 | **5.28** | >250 | >250 | >16.04 | **>32.01** |
| BRF7 | 15.62 | 15.62 | 38.13 | 45.17 | 2.44 | 2.89 | 41.39 | 68.29 | 2.65 | 4.37 | 6.84 | 17.31 | 0.44 | 1.11 | 22.09 | 41.27 | 1.41 | 2.64 | >250 | >250 | >16.04 | >16.04 |
| 109 | 15.62 | 7.81 | 38.13 | 45.17 | 2.44 | **5.78** | 41.39 | 68.29 | 2.65 | **8.74** | 6.84 | 17.31 | 0.44 | **2.22** | 22.09 | 41.27 | 1.41 | **5.28** | >250 | >250 | >16.04 | **>32.01** |
| 309 | 15.62 | 7.81 | 38.13 | 45.17 | 2.44 | **5.78** | 41.39 | 68.29 | 2.65 | **8.74** | 6.84 | 17.31 | 0.44 | **2.22** | 22.09 | 41.27 | 1.41 | **5.28** | >250 | >250 | >16.04 | **>32.01** |
| 18 | 31.25 | 7.81 | 38.13 | 45.17 | 1.22 | **5.78** | 41.39 | 68.29 | 1.32 | **8.74** | 6.84 | 17.31 | 0.22 | **2.22** | 22.09 | 41.27 | 0.71 | **5.28** | >250 | >250 | >8.00 | **>32.01** |
| 19RP | 7.81 | 7.81 | 38.13 | 45.17 | **4.88** | **5.78** | 41.39 | 68.29 | **5.30** | **8.74** | 6.84 | 17.31 | **0.88** | **2.22** | 22.09 | 41.27 | **2.83** | **5.28** | >250 | >250 | **>32.01** | **>32.01** |
| 64A | 31.25 | 15.62 | 38.13 | 45.17 | 1.22 | 2.89 | 41.39 | 68.29 | 1.32 | 4.37 | 6.84 | 17.31 | 0.22 | 1.11 | 22.09 | 41.27 | 0.71 | 2.64 | >250 | >250 | >8.00 | >16.04 |
| 71A | 62.5 | 31.25 | 38.13 | 45.17 | 0.61 | 1.45 | 41.39 | 68.29 | 0.66 | 2.19 | 6.84 | 17.31 | 0.11 | 0.55 | 22.09 | 41.27 | 0.35 | 1.32 | >250 | >250 | >4.00 | >8.00 |
| 73A | 31.25 | 31.25 | 38.13 | 45.17 | 1.22 | 1.45 | 41.39 | 68.29 | 1.32 | 2.19 | 6.84 | 17.31 | 0.22 | 0.55 | 22.09 | 41.27 | 0.71 | 1.32 | >250 | >250 | >8.00 | >8.00 |
| 1193 | 7.81 | 7.81 | 38.13 | 45.17 | **4.88** | **5.78** | 41.39 | 68.29 | **5.30** | **8.74** | 6.84 | 17.31 | **0.88** | **2.22** | 22.09 | 41.27 | **2.83** | **5.28** | >250 | >250 | **>32.01** | **>32.01** |
| 3614 | 31.25 | 15.62 | 38.13 | 45.17 | 1.22 | 2.89 | 41.39 | 68.29 | 1.32 | 4.37 | 6.84 | 17.31 | 0.22 | 1.11 | 22.09 | 41.27 | 0.71 | 2.64 | >250 | >250 | >8.00 | >16.04 |
| **NTM** |  |  |  |  |  |  |  |  |  |  |  |  |  |  |  |  |  |  |  |  |  |  |
| *M. abscessus* | 7.81 | 125 | 38.13 | 45.17 | **4.88** | 0.36 | 41.39 | 68.29 | **5.30** | 0.55 | 6.84 | 17.31 | **0.88** | **0.14** | 22.09 | 41.27 | **2.83** | 0.33 | >250 | >250 | **>32.01** | **>2.00** |
| *M. avium* | 31.25 | >250 | 38.13 | 45.17 | 1.22 | 0.18 | 41.39 | 68.29 | 1.32 | 0.27 | 6.84 | 17.31 | 0.22 | 0.07 | 22.09 | 41.27 | 0.71 | 0.17 | >250 | >250 | >8.00 | >1.00 |
| **Gram-positive** |  |  |  |  |  |  |  |  |  |  |  |  |  |  |  |  |  |  |  |  |  |  |
| *S. aureus* | 7.81 | 3.90 | 38.13 | 45.17 | **4.88** | **11.58** | 41.39 | 68.29 | **5.30** | **17.51** | 6.84 | 17.31 | **0.88** | **4.44** | 22.09 | 41.27 | **2.83** | **10.58** | >250 | >250 | **>32.01** | **>64.10** |
| *E. faecalis* | >250 | >250 | 38.13 | 45.17 | 0.15 | 0.18 | 41.39 | 68.29 | 0.17 | 0.27 | 6.84 | 17.31 | 0.03 | 0.07 | 22.09 | 41.27 | 0.09 | 0.17 | >250 | >250 | >1.00 | >1.00 |
| **Gram-negative** |  |  |  |  |  |  |  |  |  |  |  |  |  |  |  |  |  |  |  |  |  |  |
| *E. coli* | >250 | >250 | 38.13 | 45.17 | 0.15 | 0.18 | 41.39 | 68.29 | 0.17 | 0.27 | 6.84 | 17.31 | 0.03 | 0.07 | 22.09 | 41.27 | 0.09 | 0.17 | >250 | >250 | >1.00 | >1.00 |
| *M. abscessus: Mycobacterium abscessus; M. avium: Mycobacterium avium; S. aureus: Staphylococcus aureus; E. faecalis: Enterococcus faecalis; E. coli: Escherichia coli;* SI: selectivity index; J774.A1 *Mus musculus* macrophage reticulum sarcoma; VERO: *Cercopithecus aethiops* normal kidney cells; HeLa: human adenocarcinoma cells; MRC-5: human normal fibroblasts; SBC: sheep’s blood cells; IC_50_: The half-maximum inhibitory concentration; HC_50_: 50% hemolysis concentration; In bold: best results. | | | | | | | | | | | | | | | | | | | | | | |
